# Comparison of infectivity of *Plasmodium vivax* to wild-caught and laboratory-adapted (colonized) *Anopheles arabiensis* mosquitoes in Ethiopia

**DOI:** 10.1186/s13071-020-3998-2

**Published:** 2020-03-06

**Authors:** Wakweya Chali, Temesgen Ashine, Elifaged Hailemeskel, Abrham Gashaw, Temesgen Tafesse, Kjerstin Lanke, Endashaw Esayas, Soriya Kedir, Girma Shumie, Sinknesh Wolde Behaksra, John Bradley, Delenasaw Yewhalaw, Hassen Mamo, Beyene Petros, Chris Drakeley, Endalamaw Gadisa, Teun Bousema, Fitsum G. Tadesse

**Affiliations:** 1 Malaria and Neglected Tropical Diseases Directorate, Armauer Hansen Research Institute, PO Box 1005, Addis Ababa, Ethiopia; 2 Department of Microbial, Cellular and Molecular Biology, College of Natural and Computational Sciences, Addis Ababa University, PO Box 1176, Addis Ababa, Ethiopia; 3 Department of Medical Microbiology, Radboud University Medical Center, 6525 GA Nijmegen, The Netherlands; 4 Oromia Regional Laboratory, Oromia Regional Health Bureau, Adama, Ethiopia; 5 Department of Immunology and Infection, London School of Hygiene & Tropical Medicine, WC1E 7HT London, UK; 6 Tropical and Infectious Diseases Research Center, Jimma University, P.O.Box 5195, Jimma, Ethiopia; 7 Institute of Biotechnology, Addis Ababa University, PO Box 1176, Addis Ababa, Ethiopia

**Keywords:** Wild mosquito, *Anopheles arabiensis*, *Plasmodium vivax*, Membrane-feeding, Infectivity, Relative permissiveness

## Abstract

**Background:**

Mosquito-feeding assays that assess transmission of *Plasmodium* from man-to-mosquito typically use laboratory mosquito colonies. The microbiome and genetic background of local mosquitoes may be different and influence *Plasmodium* transmission efficiency. In order to interpret transmission studies to the local epidemiology, it is therefore crucial to understand the relationship between infectivity in laboratory-adapted and local mosquitoes.

**Methods:**

We assessed infectivity of *Plasmodium vivax-*infected patients from Adama, Ethiopia, using laboratory-adapted (colony) and wild-caught (wild) mosquitoes raised from larval collections in paired feeding experiments. Feeding assays used 4–6 day-old female *Anopheles arabiensis* mosquitoes after starvation for 12 h (colony) and 18 h (wild). Oocyst development was assessed microscopically 7 days post-feeding. Wild mosquitoes were identified morphologically and confirmed by genotyping. Asexual parasites and gametocytes were quantified in donor blood by microscopy.

**Results:**

In 36 paired experiments (25 *P. vivax* infections and 11 co-infections with *P. falciparum*), feeding efficiency was higher in colony (median: 62.5%; interquartile range, IQR: 47.0–79.0%) compared to wild mosquitoes (median: 27.8%; IQR: 17.0–38.0%; *Z* = 5.02; *P* < 0.001). *Plasmodium vivax* from infectious individuals (51.6%, 16/31) infected a median of 55.0% (IQR: 6.7–85.7%; range: 5.5–96.7%; *n* = 14) of the colony and 52.7% (IQR: 20.0–80.0%; range: 3.2–95.0%; *n* = 14) of the wild mosquitoes. A strong association (*ρ*_(16)_ = 0.819; *P* < 0.001) was observed between the proportion of infected wild and colony mosquitoes. A positive association was detected between microscopically detected gametocytes and the proportion of infected colony (*ρ*_(31)_ = 0.452; *P* = 0.011) and wild (*ρ*_(31)_ = 0.386; *P* = 0.032) mosquitoes.

**Conclusions:**

Infectivity assessments with colony and wild mosquitoes yielded similar infection results. This finding supports the use of colony mosquitoes for assessments of the infectious reservoir for malaria in this setting whilst acknowledging the importance of mosquito factors influencing sporogonic development of *Plasmodium* parasites.
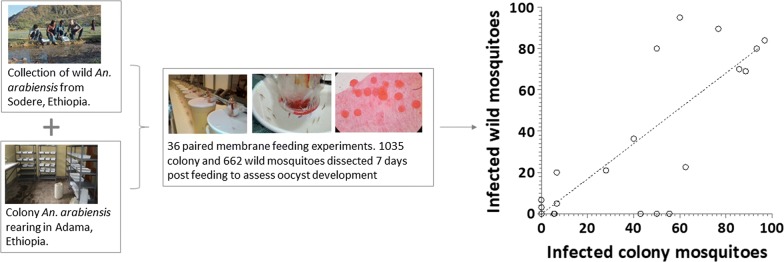

## Background

With the move towards malaria elimination and eradication, new tools and strategies to reduce onward transmission of *Plasmodium* infections, including transmission-blocking interventions (TBI), are considered highly beneficial [1, 2]. An increasing number of drug- and vaccine-based TBI are in the pipeline [3] and will require monitoring tools for efficacy. Additionally, it is considered highly beneficial to characterize the human infectious reservoir for malaria in low endemic settings approaching elimination, to better target and monitor TBI [4, 5]. Both TBI evaluation and infectious reservoir characterization require robust tools to measure human infectivity to mosquitoes. Mosquito-feeding assays can directly assess *Plasmodium* transmission from man-to-mosquitoes and play central role to estimate efficacy of TBI and the assessment of the infectious reservoir [6].

Mosquito-feeding assays allow mosquitoes to feed directly on skin of individuals (direct feeding) or on fresh human blood through an artificial membrane (membrane-feeding) [7], after which mosquito midguts are examined for parasite developmental stages (oocysts), the definitive proof that the mosquito became infected [6, 8]. Mosquito-feeding experiments are logistically demanding but increasingly used in field-based studies [5, 9–14]. Previous studies used mainly mosquitoes colonized in laboratories for several generations [5, 7, 9, 14–23]. Laboratory-adapted (colony) mosquitoes offer significant advantage over wild-caught (wild) mosquitoes in terms of logistics, ease of maintenance, flexibility of scaling-up and reproducibility of experiments [24]. However, colony mosquitoes may not fully reflect natural mosquito populations.

Maintenance of insects in artificial breeding conditions favors accumulation of traits that favor survival in the new environment, resulting in a change in genetic make-up over generations [25]. Parasite-mosquito combinations and their susceptibility to malaria infection are regulated at multiple steps during the development of the parasites [26] and numerous factors may modulate this interaction. These factors range from mosquito genetics [27, 28] and immune system [29] to parasite polymorphisms that allow evasion of the mosquito immune system [30]. Environmental factors such as midgut microbiota [31, 32], mosquito larval diet [33, 34], and temperature to support sporogony [35] are also implicated. These findings emphasize that infectivity studies from colony mosquitoes might not represent the infectivity in natural settings and therefore, assessment of the relative permissiveness of colony and wild mosquitoes could assist in the interpretation of mosquito-feeding assays to the local context. In this study, the relative permissiveness to *Plasmodium vivax* infection of colony and wild *Anopheles arabiensis* mosquitoes was assessed in paired experiments.

## Methods

### Study site, immature mosquito stages collection and rearing

Data was collected from September 2018 to February 2019 in Adama, Ethiopia (formerly called Nazareth), a city located within the Great Rift Valley, with an average elevation of ~ 1624 meters above sea level. Extensive irrigation activities characterize the area surrounding Adama with an annual peak malaria transmission season occurring between September and November [5, 36]. Both *P. falciparum* and *P. vivax* are endemic; the latter contributes towards ~ 60% of the cases [5, 37].

Immature mosquito stages (larvae/pupae) were collected by standard dipping method from potential breeding sites located at ~ 35 km from the city, close to a hot spring resort (Sodere, 8°24′N, 39°23′E, at an altitude of 1360 meters above sea level) [38]. The breeding habitat is located at a publicly accessible site where there are temporary/permanent puddles made of rock pool or pools in a grassy area (Additional file 1: Figure S1) emanating from a natural hot spring sources which exist throughout the year and form a marshy area. The collected larvae, transported in plastic jars to the field laboratory, were maintained in plastic trays in the original water collected from the breeding sites and provided with fish food (Cichlid Sticks; Tetra, Maidenhead Aquatics, Leicester, UK). Pupae were picked in glass beakers containing sedimented water from the breeding sites and kept in cages until emergence to adults. Adult female *An. arabiensis* mosquitoes were identified morphologically using standard keys [39, 40]. Colony mosquitoes (> 800th generation) were reared to adulthood as described previously [5]. Mosquitoes of both sources were maintained at the same laboratory settings; developmental stages were reared using fish food (Cichlid Sticks, Tetra) and adult mosquitoes were maintained on sucrose solution (10%) at ambient conditions at temperatures of 26–30 °C and a relative humidity of 60–80% before and after feeding.

### Membrane-feeding assays

Venous blood samples (5 ml) were collected after obtaining informed written consent from patients with microscopy-confirmed *P. vivax* infection attending the Adama Malaria Clinic. Blood collected in lithium heparin tubes (Vacutainer; BD, Oxford, UK) was offered to colony and wild *An. arabiensis* mosquitoes in parallel using membrane-feeding apparatus as detailed previously [7]. Briefly, 5–6 day-old female mosquitoes were starved for 12 h (colony) and 18 h (wild) before feeding. This timing was decided upon following pilot experiments where aggressiveness was unfavorable for wild mosquitoes after 12 h starvation. We have observed a positive association between starvation time and feeding efficiency (*ρ*_(41)_ = 0.352; *P* = 0.024); 18 h was considered appropriate for wild-caught mosquitoes with sufficient numbers of fully fed mosquitoes and minimal mortality. Feeding was performed in the dark for 25 min using water-jacketed glass-feeders (mini-feeder; Coelen Glastechniek, Arnemuiden, the Netherlands) that were covered with an artificial membrane (parafilm) and connected to a circulating water bath (Julabo GmbH; Seelbach, Germany) maintained at 38 °C. Unfed and partially-fed mosquitoes were removed from the holding cages, leaving fully-fed mosquitoes undisturbed. Fully-fed mosquitoes were maintained for 7 days under the same laboratory condition using 10% sucrose solution. At least 10 mosquitoes were dissected, and oocyst presence was assessed microscopically after staining with 1.0% mercurochrome (Sigma-Aldrich, Taufkirchen, Germany). This minimum number was mainly determined by the feeding efficiency and availability of wild mosquitoes. Asexual parasite and gametocyte densities were quantified in thick blood films, screening against 1000 leukocytes.

### Mosquito genotyping

A representative set of wild and colony mosquitoes were genotyped using multiplex polymerase chain reaction targeting the intergenic spacer gene of the ribosomal DNA of all cryptic species in the *An. gambiae* complex as described previously [41], with a few modifications. All conditions, including primers, were as per the original protocol except that the MgCl_2_ concentration was increased to 2 mM and the amplification time (at 72 °C) was raised to 40 s. Two microliters of eluate of whole mosquito body crushed in phosphate buffer saline was run in a final reaction volume of 25 µl without prior DNA extraction. In every reaction round, negative (non-template and *An. stephensi* mosquitoes) and positive controls (*An. arabiensis* colony mosquitoes) were included.

### Statistical analysis

All analyses were performed in STATA version 13 (StataCorp., TX, USA) and GraphPad Prism 5.3 (GraphPad Software Inc., CA, USA). Feeding efficiency (proportion of fully-fed mosquitoes) was compared in matched experiments using the Wilcoxon matched-pairs signed-rank test. Proportions were compared by Chi-square and Fischer’s exact tests. Differences between median parasite densities between single-species infections and co-infections were assessed using Wilcoxon rank-sum test. The bias between wild and colony mosquitoes was compared using the Bland-Altman test. The correlation between mosquito infection prevalence and gametocyte density as continuous variable was determined by Spearman’s rank correlation coefficient for colony and wild mosquitoes separately. Logistic regression was performed to compare infection status between colony and wild mosquitoes using individual mosquito data. A fixed effect for human participant was included thus taking into account the number of mosquito experiments and adjusting for correlations between mosquito observations from the same blood donor.

## Results

**Fig. 1:**
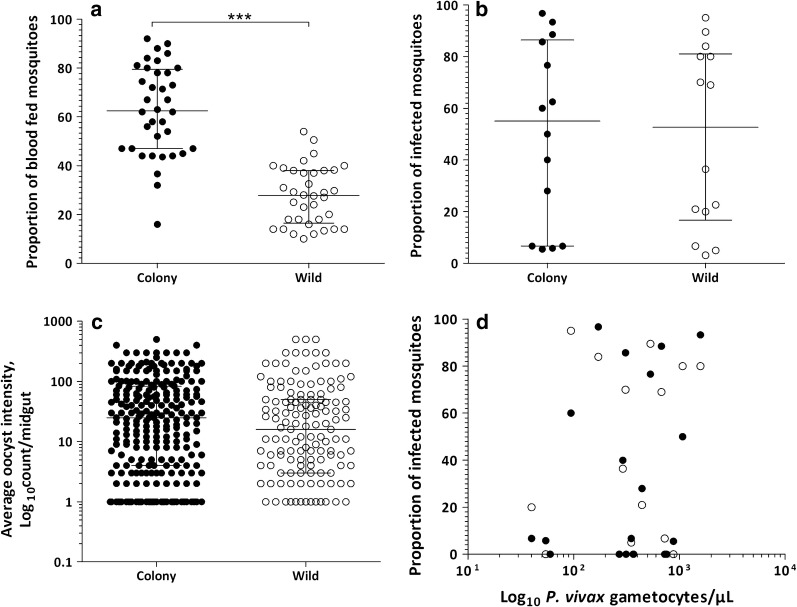
Mosquito infection outcomes in matched colony and wild *An. arabiensis* membrane-feeding experiments. The proportion of mosquitoes that were fully-fed on the patient blood through the membrane feeder (**a**) with the resulting proportion of infected mosquitoes (**b**) are indicated together with the log_10_-transformed oocyst intensity per midgut of infected mosquito (**c**) for colony (filled dots) and wild-caught (unfilled dots) mosquitoes. **d** The association between the proportion of infected mosquitoes (Y-axis) and log_10_-transformed gametocyte densities/µl (X-axis) measured by microscopy for colony (filled dots) and wild-caught (unfilled dots) mosquitoes. Lines in **a**–**c** indicate median values and 25th and 75th percentiles. The asterisks in **a** indicate a statistically significant difference between the wild and colony mosquitoes (*P* < 0.001)

A total of 36 matched membrane-feeding assays (MFA)with colony and wild mosquitoes were performed on blood samples from patients (25 *P. vivax* single- and 11 mixed-species infections with *P. falciparum*). The median age of the patients was 23.5 years (interquartile range, IQR: 18.0–29.5) and the majority of participants were male (72.2%, 26/36) (Table 1). A total of 1755 colony and 2303 wild mosquitoes were used for feeding experiments of which 1035 (59.0%) and 662 (28.7%) successfully took a blood meal, respectively. Feeding efficiency varied between colony (median: 62.5%; IQR: 47.0–79.0%) and wild (median: 27.8%; IQR: 17.0–38.0%; *Z* = 5.02; *P* < 0.001) mosquitoes (Fig. 1a). Of the total feeding experiments, 52.8% (19/36) infected at least one colony and/or wild mosquitoes. Of *P. vivax* single-species infected patients, 64.0% (16/25) were infectious to mosquitoes while 27.3% (3/11) of co-infected (*P. falciparum* + *P. vivax*) patients infected at least one mosquito (odds ratio, OR: 4.74; 95% confidence interval, CI: 1.0–22.5; *P* = 0.04). Parasite and gametocyte densities were highest in *P. vivax* infections (Table 1).

**Table 1:** Comparison between *P. vivax* single species and mixed species infections with *P. falciparum*

	*P. vivax* single-species infections^a^	*P. vivax* + *P. falciparum* co-infections^a^	Total
*N*	25	11	36
Sex, % male (*n*/*N*)	68.0 (17/25)	81.8 (9/11)	72.2 (26/36)
Age, years	23 (20–29)	25 (12–30)	23.5 (18.0–29.5)
*P. vivax* asexual parasite density	10322.5 (2847.0–181,169.0)	5540.5 (1944.5–37,900.5)	8740.8 (2173.5–24,185.3)
*P. vivax* gametocyte density^b^	332.3 (133.0–605.5)	368.3 (228.5–623.8)	358.8 (133.0–605.5)
Feeding rate, colony mosquitoes	63.0 (47.0–80.0)	62.0 (44.0–78.0)	62.5 (47.0–79.0)
Feeding rate, wild mosquitoes	29.0 (16.0–38.0)	25.0 (18.0–38.2)	27.8 (17.0–38.0)

^a^All values indicated, except sex, are median (interquartile range)

^b^Indicated only among gametocyte carriers

**Fig. 2:**
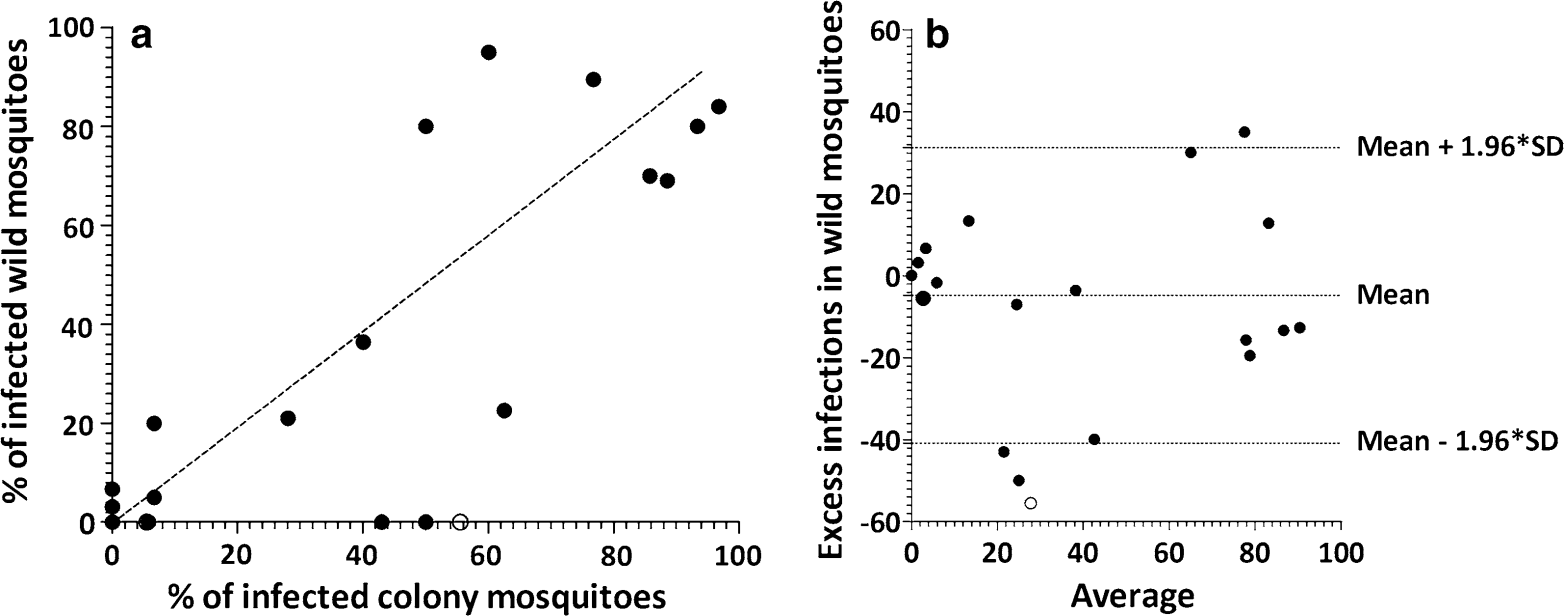
Comparison of the proportion of infected colony *vs* wild mosquitoes. **a** The proportion of infected wild mosquitoes (Y-axis) is plotted against colony mosquitoes (X-axis) for *P. vivax* single-species infections with at least 10 mosquitoes dissected. The dotted line is the line of perfect agreement. **b** The differences between the proportion of infected colony and wild mosquitoes plotted against the averages of the two mosquito sources. The average of the proportion of infected colony and wild mosquitoes for each paired infection is indicated in the X-axis *vs* excess infections in wild mosquitoes (differences between proportions of infected wild mosquitoes *vs* colony mosquitoes) in the Y-axis. The limits of agreement are indicated as the mean difference (middle dotted line) and the 95% confidence interval of the limit of agreement (mean ± 1.96 SD of differences) with horizontal dotted lines. Unfilled dots indicate *P. falciparum* + *P. vivax* co-infections

After excluding 5 matched experiments for which fewer than 10 wild mosquitoes were available for dissection, there were 31 (21 *P. vivax* single- and 10 co-infections) successful matched feeding experiments with a minimum of 10 dissected mosquitoes for both feeding approaches. In total, 66.7% (14/21) of *P. vivax* single-species infected patients infected at least one mosquito. Two patients infected either colony or wild mosquitoes; one of them infecting only colony mosquitoes but not wild (5.8% infected mosquitoes, 4/69) and *vice versa* (6.7%, 1/15). Infectious individuals infected a median of 55.0% (IQR: 6.7 – 85.7; range: 5.5–96.7%; *n* = 14) colony and 52.7% (IQR: 20.0–80.0%; range: 3.2–95.0%; *n* = 14) wild mosquitoes (Fig. 1b). The two infectious co-infected patients infected either of the colony or wild mosquitoes; one infected 5.5% (1/18) colony and the other infected 3.2% (1/32) wild mosquitoes. The median proportions of infected colony and wild mosquitoes were not different between the matched experiments (Z = 0.785; *P* = 0.433; Fig. 1b). A strong association was observed between the proportion of infected wild and colony mosquitoes (*ρ*_(16)_ = 0.819; *P* < 0.001; Fig. 2a). Overall, there was good agreement in the likelihood of becoming infected between wild and colony-reared mosquitoes. Estimation of infectivity to mosquitoes in MFAs showed no significant bias towards either mosquito source (average bias: − 4.79; 95% limits of agreement: − 40.79–31.22; *P* = 0.381; Fig. 2b). Similarly, there was no difference in the median number of oocysts (*Z* = 209; *P* = 0.835; Fig. 1c) detected in infected midguts between the colony (median: 25.0 oocysts/midgut; IQR: 5.0–83.0) and wild (median: 20.5 oocysts/midgut; IQR: 5–47) mosquitoes. In an analysis of individual mosquito data adjusted for human participant, we observed a borderline significant lower proportion of infected wild mosquitoes (OR: 0.67; 95% CI: 0.45–1.00; *P* = 0.051); plausibly reflecting differences in the number of mosquito observations in experiments.

Microscopically detected parasite density (median: 8765.5; IQR: 2199.5–26158.0; *n* = 31) was not different between patients with *P. vivax* single-species infections and patients with co-infections (*U*_(31)_ = − 103.5; *Z* = − 0.085; *P* = 0.933). Gametocytes were more frequently detected by microscopy in patients with *P. vivax* single-species infections (81.0%, 17/21) than in patients with *P. vivax* + *P. falciparum* co- infections (30.0%, 3/10; OR, 9.9; 95% CI, 1.75–56.30; *P* = 0.010) with a borderline significantly higher gametocyte density among gametocyte-positive *P. vivax* single-species infections compared to gametocyte-positive co-infections (*U*_(31)_ = − 149.5; *Z* = 1.902; *P* = 0.057). In co-infections, all microscopy-detected gametocytes were *P. vivax*. Microscopically detectable gametocyte carriers were more infectious than patients without microscopically detectable gametocytes to both colony (65.0%, 13/20 *vs* 9.1%, 1/11; OR: 18.6; 95% CI: 2.0–176.5; *P* = 0.002) and wild mosquitoes (60.0%, 12/20 *vs* 18.2%, 2/11; OR: 6.8; 95% CI: 1.1–39.8; *P* = 0.021). The proportion of infected colony (*ρ*_(31)_ = 0.452; *P* = 0.011) and wild (*ρ*_(31)_ = 0.386; *P* = 0.032) mosquitoes associated positively with gametocyte density (Fig. 1d) but not with parasite density assessed by microscopy (*ρ*_(31)_ = 0.044; *P* = 0.816) and (*ρ*_(31)_ = 0.239; *P* = 0.195), respectively. Morphologically identified wild mosquitoes were confirmed to be *An. arabiensis* using species-specific PCR for the vast majority of tested mosquitoes (96.5%; 55/57).

## Discussion

In recent years, there is increasing interest in transmission assays to evaluate TBI and assess the human infectious reservoir for malaria. More and more laboratories are establishing mosquito colonies to examine infectivity among natural infections [42]. Whilst established colonies offer some advantage in terms of feeding efficiency [43], it is generally assumed that locally relevant mosquitoes are important to allow inference to the local transmission situation. We evaluated the permissiveness of *An. arabiensis* mosquitoes raised from wild-collected larvae in comparison with colony mosquitoes maintained for over 800 generations in 36 paired MFA. Whilst mosquito feeding rates were markedly higher in colony mosquitoes, we found no evidence for epidemiologically meaningful differences in infection prevalence or infection burden between mosquito sources.

In our experiments, we encountered challenges with the aggressiveness of wild mosquitoes, exemplified by roughly two-fold lower feeding rates on the membrane for wild *versus* colony mosquitoes, which is not surprising given the selection over several hundred generations in the latter. Colony mosquitoes were maintained using rabbits as source of blood for generations in the present study. Fewer mosquito observations were available for wild mosquitoes on the day of dissection for some of the infections. This may have contributed to the borderline higher proportion of MFA resulting in at least one infected colony mosquito, simply reflecting the higher number of mosquito observations [7]. Despite this, we observed a similar proportion of infected mosquitoes among infectious feeds between colony and wild mosquitoes, in line with several other studies [24, 44]. This holds true when mosquitoes were of the same [24] or different [44] species. The F1 progeny of wild-caught *An. funestus* compared with colonized *An. coluzzii* mosquitoes [44] and similarly, colonized *An. stephensi* mosquitoes compared with their field counterpart raised from wild-caught larvae and pupae [24] were equally susceptible, when the end point was oocyst detection in the midgut.

Importantly, oocyst density was high and similar between the colony and wild mosquitoes in our study in line with previous studies on *P. vivax* that used mosquitoes of different species [11, 12, 45]. Lower oocyst densities are typically observed in *P. falciparum* [46–48]. Earlier studies also examined sporozoite prevalence and load in feeding experiments; most reporting similar levels between colonized and wild mosquitoes [15]. Similar prevalence but higher sporozoite density (but only at higher sporozoite loads) was detected in the wild mosquitoes in one of the studies [24]. Given the strong association between oocyst prevalence and intensity [49] and the strong association between oocyst density and sporozoite densities [6, 8, 50], it seems intuitive that highly similar oocyst burden, as observed in our study, precludes large differences in sporozoite density. Furthermore, variations in insectary and natural conditions that allow sporogony might potentially explain some of the differences observed [15, 24]. Mosquito innate immune responses can abrogate infections through melanization [51]. We have not observed any evidence for melanization in the present study. In addition, we also examined mosquito guts for pathogens that may influence parasite development such as microsporidia [43] and found no evidence for this. Future studies may nevertheless benefit from examining sporozoites, a limitation of the present study. Investigation of effects of environmental factors on sporogony with a specific focus on midgut microbiota that can influence transmission efficiency by stimulating the mosquito innate immune system and production of metabolites directly impairing parasite survival will also be informative [32]. In addition, mosquito blood-meal size, a poorly studied parameter that may be higher in colony- and membrane-adapted mosquitoes, needs to be considered in future evaluations. We have reared wild collected and colony developmental stages to adults at the same laboratory conditions using the same larval food to minimize the chance this could contribute to a larger body size [52] and subsequently to higher oocyst prevalence and density as a function of larger volume of blood ingested (and therefore more gametocytes) [53, 54]. Future studies would benefit by including wing length measurement as an indication of mosquito body size.

To the best of our knowledge, our findings are the first of its kind with African vivax malaria which is commonly referred to as a major cause of malaria outside sub-Saharan Africa [55]. Ethiopia forms an exception with vivax malaria, contributing towards three-quarters of the global burden together with India and Pakistan [56]. One of the unique features of *P. vivax* is the earlier generation of gametocytes, i.e. within 3–4 days after the first appearance of asexual parasites [57]. As a result, most patients start infecting mosquitoes before the onset of symptoms [58]. Despite a limited number of studies reporting a lack of association between microscopically determined gametocyte density and infectivity to mosquitoes [59], a very strong association was observed in the likelihood of infectivity between gametocyte densities and both colony and wild mosquitoes in our study. This is concordant with previous reports that used colonized *An. dirus* [9] and *An. arabiensis* mosquitoes [5] as well as *An. stephensi* [60] and *An. darligi* wild mosquitoes [61] raised from wild-collected immature stages and F1 generations, respectively.

One relevant limitation of our study was the limited sample size, relying on 36 blood donors but a total of 1755 colony and 2303 wild mosquitoes were used for the feeding experiments. We can thus not rule-out subtle differences between colony and wild mosquitoes. It would, however, be questionable whether small differences would render colony mosquitoes less suitable for assessments of the human infectious reservoir or the evaluation of interventions.

## Conclusions

The results of the present study indicate that colony mosquitoes perform at a similar level with mosquitoes caught from the wild that reflect the natural phenomenon, indicating colony mosquitoes can be used interchangeably. Our understanding of malaria transmission dynamics would benefit from similar studies in different settings with different vector and parasite species combinations, with a specific focus on mosquito determinants affecting sporogonic development.

## Supplementary information

**Additional file 1: Figure S1.** Larva and/or pupa collection habitats. Breeding habitats were mainly temporary/permanent puddles (**a–f**) or marshy (**g–h**) areas, following the streamline of a local hot spring. All the potential breeding habitats were not in use by people living close-by (within a radius of 300–500 m) and had no shading. Larvae were detected at all potential breeding sites with an average larval density of 19.5 larvae per dip. Pupae were detected at 4/9 sites where larvae were detected during a single visit. The median volume of the breeding habitat was 0.20 m^3^ (IQR: 0.08–0.57 m^3^; range: 0.004–7.50 m^3^).

## Data Availability

Data supporting the conclusions of this article are provided within the article and its additional file. Raw data will be made available upon request.

## Abbreviations

TBI: transmission-blocking interventions
MFA: membrane feeding assay
