# Supplementary figures and images for: Comparison of infectivity of Plasmodium vivax to wild-caught and laboratory-adapted (colonized) Anopheles arabiensis mosquitoes in Ethiopia
Source: Parasit Vectors. 2020 Mar 6;13:120. doi: 10.1186/s13071-020-3998-2 (PMC7059271; doi:10.1186/s13071-020-3998-2)

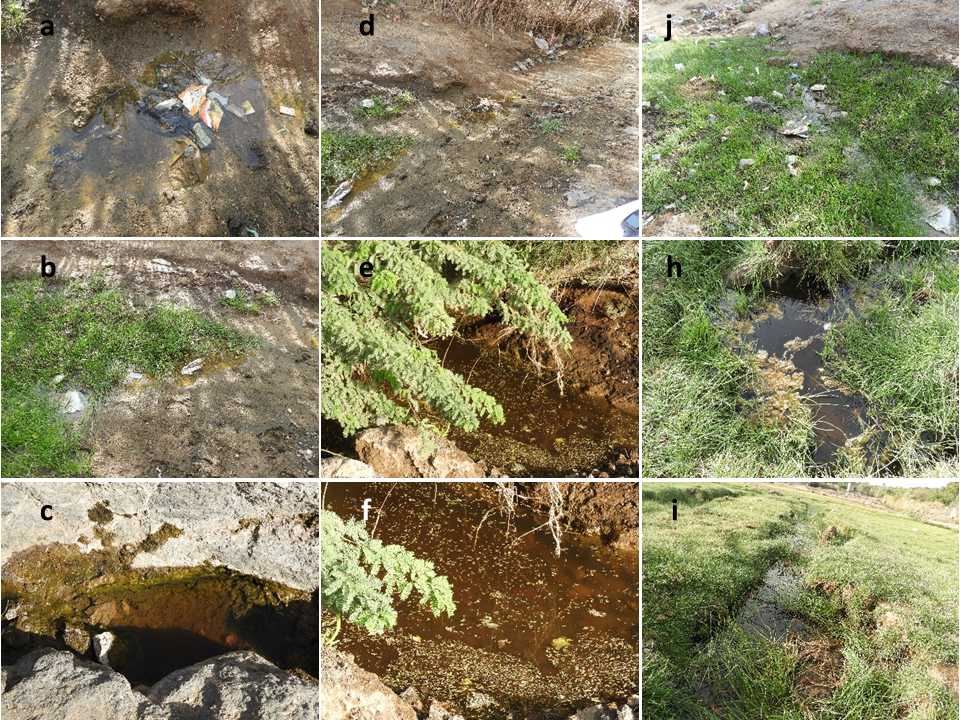

Supplement: Supplementary file 1 — Additional file 1: Figure S1. Larva and/or pupa collection habitats. Breeding habitats were mainly temporary/permanent puddles (a–f) or marshy (g–h) areas, following the streamline of a local hot spring. All the potential breeding habitats were not in use by people living close-by (within a radius of 300–500 m) and had no shading. Larvae were detected at all potential breeding sites with an average larval density of 19.5 larvae per dip. Pupae were detected at 4/9 sites where larvae were detected during a single visit. The median volume of the breeding habitat was 0.20 m3 (IQR: 0.08–0.57 m3; range: 0.004–7.50 m3). [file 13071_2020_3998_MOESM1_ESM.tif]
